# Persistent Difficulties in Switching to Second-Line ART in Sub-Saharan Africa — A Systematic Review and Meta-Analysis

**DOI:** 10.1371/journal.pone.0082724

**Published:** 2013-12-23

**Authors:** Yoann Madec, Sandrine Leroy, Marie-Anne Rey-Cuille, Florence Huber, Alexandra Calmy

**Affiliations:** 1 Institut Pasteur, Emerging Diseases Epidemiology Unit, Paris, France; 2 SOLTHIS, Paris, France; 3 Geneva University Hospital, HIV Unit, Department of Internal Medicine, Geneva, Switzerland; University of Washington, United States of America

## Abstract

**Objectives:**

Switching to second-line antiretroviral therapy (ART) largely depends on careful clinical assessment and access to biological measurements. We performed a systematic review and meta-analysis to estimate the incidence of switching to second-line ART in sub-Saharan Africa and its main programmatic determinants.

**Methods:**

We searched 2 databases for studies reporting the incidence rate of switching to second-line ART in adults living in sub-Saharan Africa. Data on the incidence rate of switching were pooled, and random-effect models were used to evaluate the effect of factors measured at the programme level on this incidence rate.

**Results:**

Nine studies (157,340 patients) in 21 countries were included in the meta-analysis. All studies considered patients under first-line ART and conditions to initiate ART were similar across studies. Overall, 3,736 (2.4%) patients switched to second-line ART. Incidence rate of switch was in mean 2.65 per 100 person-years (PY) (95% confidence interval: 2.01–3.30); it ranged from 0.42 to 4.88 per 100 PY and from 0 to 4.80 per 100 PY in programmes with and without viral load monitoring, respectively. No factors measured at the programme level were associated with the incidence rate of switching to second-line ART.

**Conclusion:**

The low incidence rate of switching to second-line ART suggests that the monitoring of patients under ART is challenging and that access to second-line ART is ineffective; efforts should be made to increase access to second-line ART to those in need by providing monitoring tools, education and training, as well as a more convenient regimen.

## Introduction

The number of patients on antiretroviral therapy (ART) has dramatically increased by more than 26-fold between 2003 and 2011 in resource-limited settings [1], where ART has been proven to be as successful as in developed countries with regards to clinical, immunological or virological outcomes [2]–[5]. However, a first ART (first-line) may fail, and tools to detect therapeutic failure differ between countries; viral load testing is the gold standard to inform the switching decision to a more successful regimen in wealthy countries [2]. The World Health Organisation (WHO) recognises that definitions and tools for the detection and management of treatment failure are not standardized and has outlined a set of definitions for treatment failure, including immunological and clinical criteria, to be used with or without virological criteria. A number of observational studies have found that clinical markers alone or in combination with immunological status, as recommended by the WHO, poorly predict virological failure [3], [4], [5], [6], [7]. If clinical trials failed to demonstrate that viral load monitoring translated to survival gain [8], it remains that in the absence of routine viral load, detection of treatment failure and the subsequent switch to second-line ART usually occurred late. Moreover, patients who continue on a failing regimen tend to accumulate drug resistance mutations over time [9], [10], resulting in increased mortality [11] and lower risk of future virological suppression [12]. In addition, HIV transmission is more likely to occur due to on-going viral replication.

Our aim is to describe access to second-line ART in sub-Saharan Africa. With this meta-analysis, we estimated the incidence rate of switching to second-line ART in sub-Saharan Africa and evaluated the effect of factors measured at the programme level on this incidence rate.

## Methods

We performed a systematic review and meta-analysis to estimate the incidence rate of switching to second-line ART in sub-Saharan Africa and to search for influencing effects, in accordance with the Centre for Reviews and Dissemination guidelines [13] and standards of reporting for systematic reviews (PRISMA) [14].

### Search Strategy

Studies were searched for using PubMED (last update: 22/03/2012) and Embase (last update: 12/06/2012) using the following keywords in the text form: (ART OR HAART OR “antiretroviral”) AND (Africa OR “Sub Saharan” OR “resource limited” OR “resource-limited” OR “low resource” OR “resource poor” OR “resource-constrained”) AND (Switch OR “Switched” OR modification OR “treatment changes” OR “second line”). This computerized search was completed with a manual review of the reference lists of the articles, without language restriction.

### Study Eligibility and Inclusion

Published studies that provided incidence rate of switching to second-line ART in adults (as defined in each study, and ranged from ≥15 years to ≥18 years) in sub-Saharan Africa in either observational cohort studies or clinical trials were eligible to enter our meta-analysis. We defined the incidence rate as the number of switches to second-line ART divided by the cumulative number of person-years of follow-up. We therefore included all the studies which provided either the incidence rate of switching to second-line ART, or the total number of switches and the total duration of follow-up. We excluded studies that reported only the number of patients on second-line ART or the cumulative incidence of switch to second line ART (expressed as the percentage of patients under second-line ART) without mention of follow-up duration, as they did not allow to estimate the incidence rate. Studies were eligible in our meta-analysis regardless of the number of patients enrolled. We also included studies without condition on a minimum patient’s follow-up on ART; i.e. we considered studies which enrolled patients without condition on their minimum duration of follow-up as well as studies which only enrolled patients who had reached a minimum duration of follow-up. We further excluded all studies taking place in another region, or exclusively addressing children or reporting all treatment modification without specifically reporting the number of switches to second-line ART. Studies reporting incidence rate of switching to second-line ART based on mathematical models were also excluded.

In all the studies considered, a switch to second-line ART was defined as the introduction of a protease inhibitor (PI) in place of the non nucleoside reverse transcriptase inhibitor (NNRTI), with or without change of the nucleoside reverse transcriptase inhibitor (NRTI) backbone.

### Data Identification and Extraction

Two of the authors (YM and MARC) independently assessed the title and abstract of each potential study from the electronic search and rejected it if it was clearly ineligible. All eligible articles were then fully and independently reviewed against inclusion/exclusion criteria by the two authors. At both steps (eligibility and final inclusion), disagreements were resolved by discussion and consensus.

The following information was extracted from the articles included in the meta-analysis: type of study (observational or clinical trial), type of site (public sector, NGO-operated, clinical trial setting), year of publication, country (or countries) where the study took place, period of enrollment, number of patients, number of patients who switched to second-line ART, total duration of follow-up in person-years and/or incidence rate of switching to second-line ART, proportion of women, median age at ART initiation, median CD4 count at ART initiation, proportion of patients in WHO clinical stage 3 or 4 at ART initiation, proportion of patients for each ART combination, and availability of viral load testing.

Quality of the studies considered in this meta-analysis was assessed through a pre-defined instrument based on the STROBE (strengthening the reporting of observational studies in epidemiology) checklists [15]. Availability of information regarding the description of the setting, the reporting of key dates, the source of the data, the eligibility criteria, the definition of the main outcome of the study, the presentation of baseline characteristics of the patients, the reporting of the main outcome unadjusted and after adjustment for the main cofactors, and the discussion of the limitations of the study was checked for all studies.

### Statistical Analysis

We used a random effect model, without weight using the *metan* command from the Stata software (Stata Corp, College Station, TX) to estimate the overall incidence rate of switching to second-line ART, based on incidence rate estimates and 95% lower and upper confidence interval limits estimated through a Poisson model. Heterogeneity is indicated if the Cochran test is significant at the level 0.05 and/or the I-square estimator is above 0.50.

To estimate the influence of the programmes’ characteristics on the incidence rate of switching to second-line ART, we conducted a meta-regression analysis considering the following factors: proportion of women, proportion of patients in WHO stage 3 or 4, median CD4 level, mean duration of follow-up (all dichotomized based on the median), year of beginning of follow-up (≤2002, 2003, ≥2004), availability of viral load monitoring (routine or performed on demand versus not available), the type of site (public sector, NGO-operated, or clinical trial setting), previous exposure to ART (studies strictly enrolling ART-naïve patients versus the other studies), first-line ART (EFV-based or NVP-based) and the definition of second-line ART (PI introduction only or PI introduction with modification of at least 1 NRTI) on the incidence of switching to second-line ART.

A sensitivity analysis was conducted excluding randomized trials as the follow-up in such studies does not usually correspond to the routine care in sub-Saharan Africa.

## Results

### Selection of Studies

**Figure 1:**
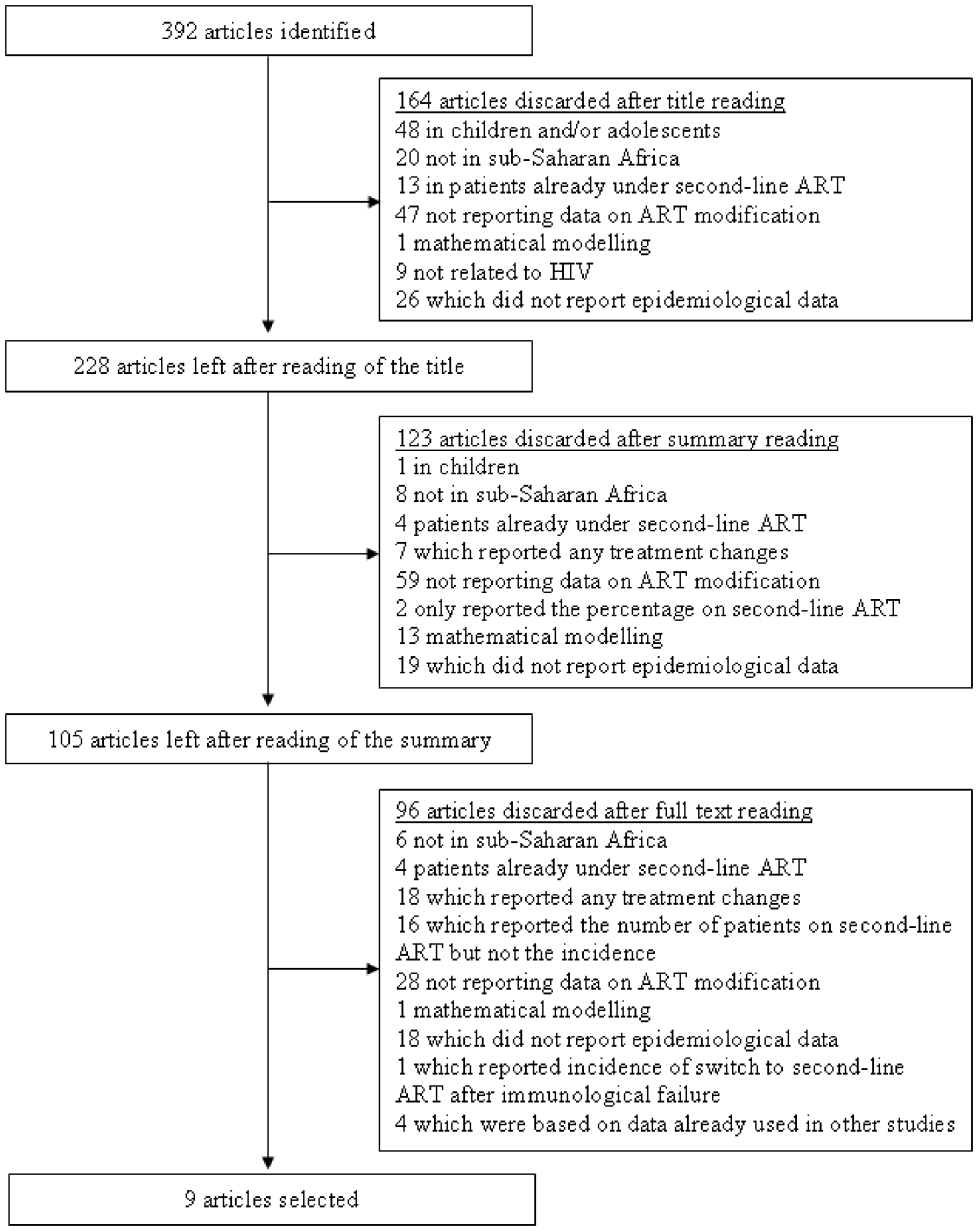
Flow diagram of study selection process.

The search on PubMED and Embase produced 392 studies (Figure 1), of which 164 were excluded based on the title only (Figure 1). Another 123 studies were excluded based upon the abstract, leaving 105 published papers for full text screening.

Of these 105 articles, nine were included in our meta-analysis because they reported the number of switches to second-line ART and the total duration of follow-up in person-years (PY) [8], [16], [17], [18], [19], [20], [21], [22], [23]. Out of these nine studies, two international studies written by the same first author had sites in common [20], [23], but the author provided the data from the oldest study (which also presented the lower number of patients) once the potential duplicates (i.e., patients enrolled in the sites in common in both studies) were removed. Thus we could consider both studies in our meta-analysis.

The remaining 96 studies were discarded for the following reasons: 16 studies that reported the number of switches to second-line ART but not the total duration of follow-up thus not enabling us to estimate the incidence rate of switching to second-line ART, four [24], [25], [26], [27] duplicated data reported in other studies [22], [24], [27], one provided the incidence of switching to second-line ART only from the time of immunological failure [32], and the 91 others for other reasons presented in Figure 1.

To evaluate the qualities of the nine studies considered in this meta-analysis, we considered some of the items from the STROBE statement which we considered relevant (Table 1).

**Table 1:** Checklist of items retained from the STROBE statement.

	Orrellet al. [16]	Pujades-Rodriguezet al. [17]	Palombiet al. [18]	DART trialteam [19]	Keiseret al. [20]	Landieret al. [21]	Auldet al. [22]	Keiseret al. [23]	Laurentet al. [8]
**Setting**									
Provides location	✓		✓	✓	✓	✓	✓	✓	✓
Provides recruitmentdates and follow-up	✓	✓		✓		Provided onrequest	✓		✓
**Participants**									
Provides eligibilitycriteria	✓	✓	✓	✓	✓	✓	✓	✓	✓
Provides the numberof participants	✓	✓	✓	✓	Provided on request	✓	✓	✓	✓
Provides the characteristics ofparticipants atenrolment	✓	✓	✓	✓	Provided on request	✓	✓	✓	✓
Summarizes follow-up time	✓	Provided onrequest	✓	✓	Provided on request	✓	✓	✓	Provided on request for one of the two study arms
**Outcome data**									
Provides a definitionof switchingto second-line ART	✓	✓	✓	✓	✓	✓	Defined by referringto Keiser et al.	✓	✓
**Discussion**									
Presents limitationsof the study		✓		✓	✓			✓	✓

### Characteristics of the Studies

The 9 studies considered in this meta-analysis accounted for 157,340 patients (Table 2) Two of these studies were clinical trials which aimed to compare two care strategies (with and without biological monitoring) and thus accounted for two incidence rates each reported as two independent studies [8], [19]. One observational cohort study comparing programmes with and without access to viral load monitoring [23] also accounted for two incidence rates each reported as two independent studies. The other studies were observational cohorts, and each provided one overall incidence rate of switching to second-line ART. Thus, these 9 studies accounted for 12 incidence rates of switching to second-line ART in adults in sub-Saharan Africa.

**Table 2:** Characteristics of the studies presenting incidence rate of switching to second-line ART.

	Countries	Type of study	N	Minimumfollow-up	Definition of switchingto 2^nd^ line ART	Number ofswitches	Incidence rate (95% CI)per 100 person-years
Orrell et al. [16]	South Africa	Observational	929	≥2 visits	PI introduction	36	4.74 (3.32–6.56)
Pujades-Rodriguezet al. [17]	Benin, Burkina Faso,Burundi, Cameroon,Ivory coast,Ethiopia, Guinea, Kenya,Malawi, Mozambique,Nigeria, Democratic Republicof the Congo, Tanzania,Uganda, Zambia, Zimbabwe	Observational	37,918	6 months	PI introduction withmodification of atleast 1 NRTI	250	0.42 (0.37–0.48)
Palombi et al. [18]	Guinea-Conakry,Malawi,Mozambique	Observational	3,749	≥2 visits	PI introduction	222	4.88 (4.26–5.57)
DART trial team –LCM [19]	Uganda, Zimbabwe	Randomized trial	1,656	≥2 visits	PI introduction	361	4.79 (4.31–5.32)
DART Trial Team –CDM [19]	Uganda, Zimbabwe	Randomized trial	1,660	≥2 visits	PI introduction	314	4.24 (3.78–4.74)
Keiser et al. [20]	Côte d’Ivoire, Kenya,Malawi, Uganda, Rwanda,Senegal, South Africa,Zambia, Zimbabwe	Observational	7,865	6 months	PI introduction withmodification of at least 1 NRTI	208	2.70 (2.35–3.09)
Landier et al. [21]	Mali	Observational	865	≥2 visits	PI introduction	40	3.27 (2.33–4.45)
Auld et al. [22]	Mozambique	Observational	2,596	≥2 visits	PI introduction withmodification of atleast 1 NRTI	24	0.69 (0.44–1.03)
Keiser et al. –With viral loadmonitoring [23]	Malawi, South Africa,Zambia	Observational	18,706	≥2 visits	PI introduction withmodification of atleast 1 NRTI	899	3.29 (3.27–3.32)
Keiser et al. –No viral loadmonitoring [23]	Malawi, South Africa,Zambia	Observational	80,937	≥2 visits	PI introduction withmodification of atleast 1 NRTI	1,369	0.93 (0.92–0.94)
Laurent et al. –LCM arm [8]	Cameroon	Randomized trial	221	≥2 visits	PI introduction	13	3.60 (1.92–6.16)
Laurent et al. –CDM arm [8]	Cameroon	Randomized trial	238	≥2 visits	PI introduction	0	0.00 (0.00–0.01)

NA: not available.

Overall, patients originating from 21 countries in sub-Saharan Africa were considered. Four studies took place in a single country [8], [16], [21], [22], and the others analyzed data from 2 to 16 countries. The minimum follow-up under first-line ART required 6 months in two studies [17], [20], 3 months in another study [21], and no restriction in the remaining studies. All but one studies considered in our meta-analysis took place in a single type of site: NGO-supported site [20], [21], randomized clinical trial site [8], [19] or public sector site [18], [21], [22], [23]. One study took place in both the public sector and NGO-operated sites [24]. Five studies only enrolled patients who were ART-naïve [8], [17], [19], [20], [23] while the other studies did not specifically mention only enrolling ART-naïve patients; however, these latter studies were based on programmes providing access to ART and a vast majority of patients are likely to be ART-naïve.

In all studies, ART initiation criteria followed WHO recommendations, and all patients initiated an NNRTI-based first-line ART (table 3). In the DART trial, the NRTI-backbone was lamivudine (3TC) and zidovudine (AZT) in all patients [19], while in the other studies the NRTI-backbone was 3TC and didanosine (d4T) in the majority of patients (range 53.1% to 90.5%) [8], [16], [17], [18], [20], [21], [22], [23] (Table 2). Regarding the NNRTI, a majority of patients received tenofovir (TDF) in the DART trial [19], efavirenz (EFV) in two studies [16], [23] and nevirapine (NVP) in the remaining studies [8], [17], [18], [20], [21], [22], [23] Second-line ART was defined as a PI introduction in the ART regimen, but in four studies, the modification of at least one NRTI molecule was also required [17], [20], [22], [23]. Overall, patients from the studies considered in this analysis initiated first-line ART from 2001 to 2008, and the end of the follow-up ranged from 2005 to 2008 (table 4).

**Table 3:** Characteristics of the studies presenting incidence rate of switching to second-line ART.

	% under 3TC-d4T-EFV	% under 3TC-d4T- NVP	% under 3TC-AZT-EFV	% under 3TC-AZT- NVP
Orrell et al. [16]	84.4	3.7	1.3	10.6%
Pujades-Rodriguez et al. [17]	NA	NA but in majority here	NA	NA
Palombi et al. [18]	NA	65.1	NA	31.4
DART trial team - LCM [19]	0	0	0	16.0
DART Trial Team - CDM [19]	0	0	0	16.0
Keiser et al. [20]	16.3	56.8	10.7	14.3
Landier et al. [21]	4.3	86.2	1.6	3.2
Auld et al. [22]	9.4	78.5	0.5	10.2
Keiser et al. –With viral loadmonitoring [23]	65.4	22.5	5.1	6.5
Keiser et al. – No viral loadmonitoring [23]	6.1	58.0	3.4	31.4
Laurent et al. – LCM arm [8]	17.0	68.0	9.0	5.0
Laurent et al. – CDM arm [8]	18.0	64.0	8.0	9.0

**Table 4:** Characteristics of the studies presenting incidence rate of switching to second-line ART.

	Year of beginning of follow-up	Year of end of follow-up	% women	Median age at baseline	Median CD4 count at baseline	% with WHO stage4 condition atbaseline	% with WHO stage3–4 conditionat baseline	Viral loadmonitoring
Orrell et al. [16]	2002	2005	72.2	33	95	27.5	81	Every 4 months
Pujades-Rodriguez et al. [17]	2001	2006	NA	NA	NA	NA	NA	Targeted
Palombi et al. [18]	2002	2007	62.0	34	192	NA	37	Every 6 months
DART trial team - LCM [19]	2003	2008	66.0	36	86	23	78	None
DART Trial Team - CDM [19]	2003	2008	64.0	36	86	24	81	None
Keiser et al. [20]	1998	NA	72.0	35	128	NA	50.5	Routine in 3 of 7 programmes (9.1% of all patients)
Landier et al. [21]	2003	2008	62.1	34	124	23	84	None
Auld et al. [22]	2004	2008	60.7	34	155	15	60	None
Keiser et al. –With viral load monitoring [23]	NA	NA	65.7	35	93	NA	57.7	Every 3–6 months
Keiser et al. – No viral load monitoring [23]	NA	NA	61.8	36	132	NA	70.7	Limited access**
Laurent et al. – LCM arm [8]	2006	2010	71	37	182	26	100	Every 6 months
Laurent et al. – CDM arm [8]	2006	2010	70	36	179	26	99	None

** as stated in the article.

NA: not available.

The mean duration of follow-up ranged from 12 to 22 months in all but two studies. In one study, the mean duration of follow-up was only 9.8 months [16], whereas in both arms of the DART trial, the mean duration of follow-up was as long as 54 months [19].

CD4 cell count was not available in the clinical-monitoring arm of both trials [8], [19], whereas in all other studies, it was routinely performed every 3 to 6 months. Viral load monitoring was not available in the clinical-monitoring arm of both trials [22], [27], in the laboratory and clinical monitoring of the DART trial [19] and in two other studies [21], [23]; it was available on demand [17] or in routine (i.e., every 3 to 6 months depending on the studies; Table 4) [16], [18], [23] in other studies. One study considered sites with and without viral load monitoring, however only 9.1% of the patients were followed in a site with virological monitoring [20].

The proportion of women was homogeneous (range: 61.8% to 72.2%) and the median age at ART initiation ranged from 33 to 37 years across studies (table 4). Overall, baseline CD4 cell count was available in 75.1% of the patients (range between studies: 64.1%–100%), without considering one multicentre study as information specific to patients from sub-Saharan Africa could not be retrieved [17]. Patients were generally at an advanced stage of the disease at ART initiation: the median CD4 count at ART initiation was below 150 cells/mm^3^ in 7 out of 11 cases (range: 86 to 192 cells/mm^3^), and the proportion of patients in WHO stage 3 or 4 at ART initiation ranged from 37.1% to 100%.

### Incidence Rate of Switching to Second-line ART

**Figure 2:**
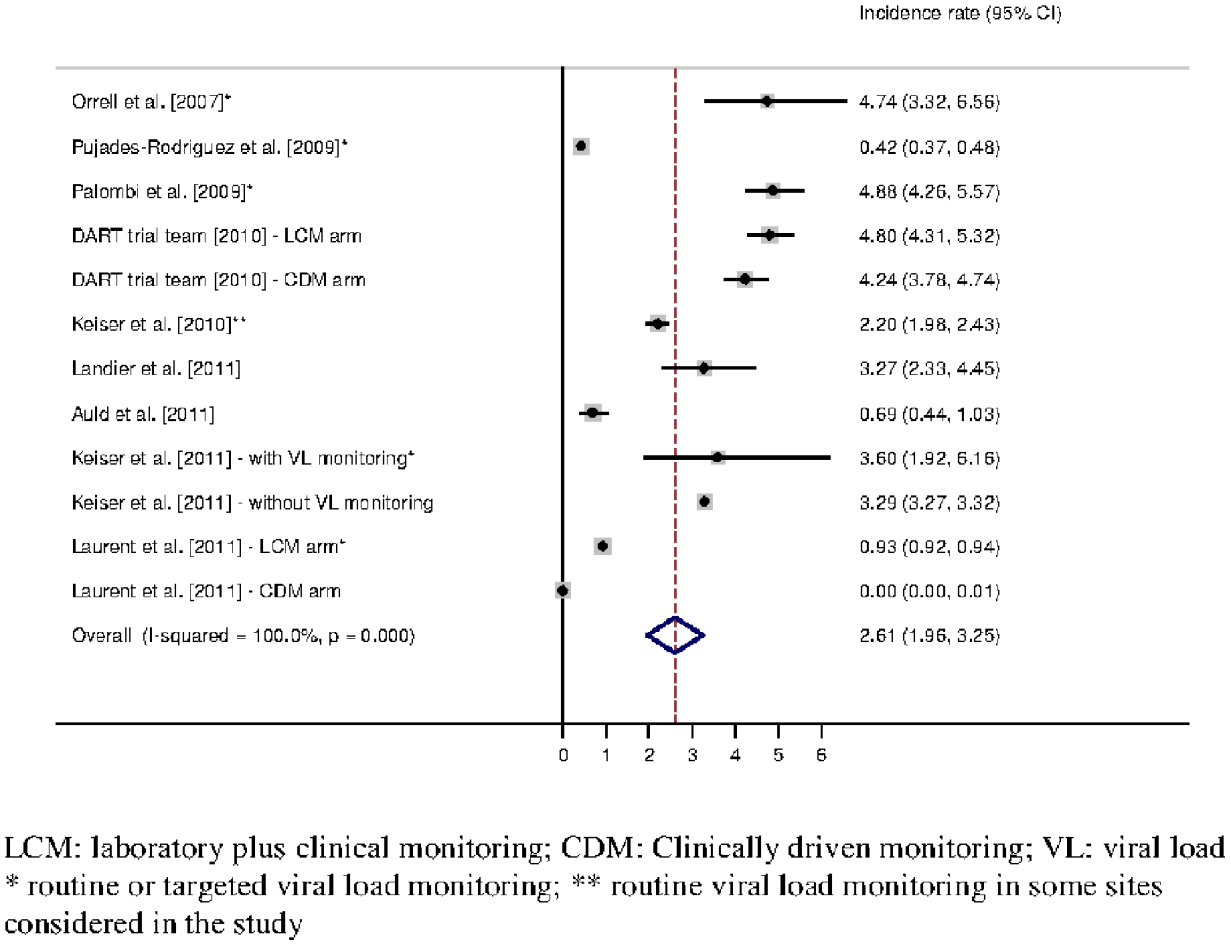
Incidence rate of switching to second-line ART (expressed per 100 person-years) – Estimation from 9 studies providing 11 incidences of switching to second-line ART.

The 157,340 patients accounted for 260,631.5 person-years (PY) of follow-up under first-line ART, and 3,736 (2.4%) patients switched to second-line ART. The overall incidence rate of switching to second-line ART, estimated from the 12 independent incidence rates, was 2.65 per 100 PY (95% confidence interval (CI): 2.01–3.30) (figure 2). The Cochran test (p< = 0.001) and the I-square (100.0%) indicated a large between-studies heterogeneity regarding incidence rate of switching to second-line ART. Of the 12 identified incidence rates of switching to second-line ART, only three studies had confidence intervals overlapping with the overall incidence rate of switching to second-line ART obtained from the meta-analysis. However, eight incidences were confined to the interval between 2 and 5 per 100 PY (figure 2).

We investigated whether characteristics measured at the programme level could influence the incidence rate of switching to second-line ART and thus explain the heterogeneity. Due to estimation problems, the clinically driven monitoring arm of the Stratall trial could not be considered in the following analysis, as no switches were reported in that arm [8]. The proportion of women in the programme (≤ or >66%; p = 0.74), the median CD4 level at ART initiation (≤ or >128 cells/mm^3^; p = 0.10), the proportion of patients in WHO stage 3–4 at ART initiation (≤ or >78%; p = 0.33), the year of beginning of the study (≤2002, 2003 or ≥2003; p = 0. 47 and p = 0.52), the mean duration of follow-up in the study (≤ or >17 months; p = 0.63), the type of sites considered (public sector, clinical trial setting, or NGO-operated; p = 0.28 and p = 0.89), pre-exposure to ART (studies strictly enrolling ART-naïve patients versus the other studies; p = 0.57), and first-line ART initiated (EFV-, NVP-or TDF-based; p = 0.95 and p = 0.30) were not associated with the incidence rate of switching to second-line ART. On the other hand, the definition of second-line ART (PI introduction only or PI introduction and modification of at least 1 NRTI; p = 0.03) was associated with the incidence rate of switching to second-line ART. Considering the 7 studies in which a second-line was defined as a PI introduction, the overall incidence rate (95% CI) of switching to second-line ART was 3.64 (1.25–6.02) per 100 PY, while it was 1.60 (0.25–3.00) per 100 PY when a change of at least one NRTI was required.

The incidence rate of switching to second-line ART was not significantly different between programmes with routine or targeted viral load monitoring on the one hand [8], [16], [17], [18], [23], and programmes without viral load monitoring on the other hand [8], [19], [20], [21], [22], [23] (p = 0.81); in this comparison the study considering programmes with and without viral load monitoring together, but where only 9.1% of the patients were followed in a programme with virological monitoring [20], was classified as without virological monitoring. The incidence rate of switching to second-line ART ranged from 0.42 to 4.88 in programmes with viral load monitoring, which led to an overall incidence rate of switching to second-line ART of 3.33 (95% CI: 1.48–5.17) per 100 PY. In programmes without access to viral load monitoring, the incidence rate of switching to second-line ART ranged from 0 to 4.80, which led to an overall incidence rate of 2.60 (95% CI: 0.94–4.26) per 100 PY.

A sensitivity analysis was conducted excluding results originating from clinical trials, as the follow-up, care and availability of second-line molecules might differ from the other settings. The incidence rate (95% CI) of switching to second-line ART was 2.53 (1.44–3.63) per 100 PY, which is close to the result obtained in the main analysis, with a slightly wider confidence interval. Both the Cochran test and I-square showed heterogeneity between studies (p<0.001 and 100%, respectively). In this sensitivity analysis, the incidence rate of switching to second-line ART tended to be lower in programmes where a switch was defined as a PI introduction along with a change of at least one NRTI than in programmes where a switch was defined solely as a PI introduction (p = 0.09). The other factors measured at the programme level were not associated with the incidence of switching to second-line ART, including the availability of viral load monitoring (p = 0.51).

## Discussion

This systematic review and meta-analysis led to an estimated incidence rate (95% CI) of switching to second-line ART of 2.65 (2.01–3.30) per 100 PY; this result was based on 12 incidence rates that were mostly between 2 and 5 per 100 PY.

Most studies considered in the meta-analysis enrolled patients in the public sector, whereas some studies took place in sites which were totally or partially NGO-operated [17], [18], [20], and two studies were clinical trials [8], [19]. However, neither the context of clinical trial nor the humanitarian aid seemed to affect the incidence rate of switching to second-line ART in these published studies. We did not find evidence that the incidence rate of switching to second-line ART was different between programmes where the majority of patients initiated an EFV- or NVP-based first-line ART, neither did we find evidence that the incidence rate was different in programmes with lower median CD4 count at ART initiation or with a higher proportion of patients in WHO stage 3 or 4.

The incidence rate of switching to second-line ART was lower in studies where at least one NRTI had to be changed. We believe that these two definitions were chosen, based on study teams’ experience, to identify switches to second-line among all treatment modifications recorded in the databases. We do not expect these two definitions to represent a difference in clinical practice.

The incidence rate of switching to second-line ART was not different in programmes with and without access to viral load monitoring. In our meta-analysis, the incidence rate of switching to second-line ART ranged essentially on the same scale whether routine viral load monitoring was available (0.42 to 4.88 per 100 PY) or not (0 to 4.80 per 100 PY). The mean follow-up time ranged essentially from 12 to 20 months; thus, we essentially estimated the incidence rate of switching during the first few years following ART initiation. This may explain the absence of effect between programmes with and without viral load monitoring.

The estimated incidence rate of switching to second-line ART of 2.65 per 100 PY seems low. However, one can wonder: what is the reasonable incidence rate of switching? To judge whether this incidence rate of switching is low, it should be put in relation with the rate of treatment failure.

### Do All Treatment Failures Require a Treatment Change?

Whereas recommendations from wealthy countries defined virological failure as the inability to achieve or maintain suppression of viral replication below a threshold of 200 copies/ml, such a level of viral replication does not automatically prompt a change to second-line ART in resource-limited settings. Consolidated WHO guidelines very recently published are to switch to second-line ART if, after an intervention to improve adherence has been implemented, the viral load remains above the threshold of 1,000 copies/ml [28].

In the absence of virological monitoring, the WHO recommends clinical and immunological criteria to identify patients who may fail under a first-line ART [29]. However, several studies showed that among these patients who presented clinical or immunological failure, only a small proportion presented viral load above thresholds defining virological failure [3], [5], [6], [7]. If targeted viral load measurement in these patients is of great interest to prevent unnecessary switches, those who do not experience clinical or immunological failure are not necessarily in virological success. Worryingly, the immunological criteria were found to have an extremely poor sensitivity, ranging from 12% of 58% [4], [6], [7]. In other words, a large proportion of patients in virological failure present no signs of clinical and/or immunological failure, while they well may need to change ART.

This finding obviously calls for a wider access to viral load monitoring in resource-limited settings.

### What is the Gap between the Determination of a True Virological Failure (Harbouring Resistance Mutations) and Treatment Change?

In a recent WHO report, the proportion of patients with virological failure at 12 months of ART was 9.4%, among whom 72.1% carried resistance to at least one HIV drug (i.e., a failure rate of 6.8% at 12 months) [1]. In a meta-analysis documenting the rate of acquired drug resistance in resource-limited settings, the proportion of patients exhibiting drug resistance was 11.1%, 15.0% and 20.7% at 12–23 months, 24–35 and ≥36 months, respectively [30]. In studies conducted in sub-Saharan Africa, when genotyping of the virus to identify drug resistances was conducted in patients with viral load >1,000 copies/ml at 12 months, the rate of failure varied across studies: 5% [31], 10% [32], [33] or even as high as 24.5% [34].

These studies suggest a therapeutic failure rate approximately 5–10% at 12 months after ART initiation, which is at least twice the incidence rate of switching to second-line ART found at 2.65 per 100 PY (95% CI: 2.01–3.30). We believe that the number switched to second-line only represents a minority of those in need for treatment change, regardless of the definition used for treatment failure.

We therefore believe that this incidence rate of switching is too low, thus jeopardising success of ART in the long run. Indeed, if patients are maintained under a failing first-line ART after undiagnosed failure, they are at high risk of resistance acquisition [3], [9], [35]. Despite positive outcomes of patients on second-line ART [36], [37], some recent evaluations of patients under second-line ART have shown high early mortality [38] and high failure rates [39], [40], which are possibly linked to a delayed switch to second-line ART after first-line treatment failure.

The low incidence rate of switching to second-line ART may be related to the well-known gap in accessing virological tools [41]; hopefully, point-of-care technology will be able to address the shortage of virological monitoring. One can also hypothesize that the cost and difficulties of taking PI-containing ART could impair both the clinician’s and the patient’s compliance to second-line ART. In addition, access to third-line ART is still scarce in resource-limited settings. In such contexts, clinicians might be reluctant to switch patients who are clinically well to second-line ART, as they may see second-line ART as a salvage therapy to be saved for later when no other option might be available [42].

### Strength and Limitations

By strictly selecting studies that reported incidence rate of switching to second-line ART, we worked on an estimator that not only considered the number of switches observed but also considered this number of switches in relation to the duration of follow-up. The drawback of this selection is that the number of studies included in our meta-analysis was fairly limited. Indeed, 16 studies that reported the number of switches to second-line ART but not the incidence rate were discarded. In those 16 studies, the mean proportion of patients switched to second-line was 4.0% (range: 0.1–21.0). For the 9 studies in our meta-analysis that reported incidence rate of switching to second-line ART, the mean proportion of patients who switched to second-line ART was 5.9% (range: 0.0–21.8). These proportions were similar. We therefore do not expect that discarding the 16 studies that only reported the number of switches to second-line ART affected our results.

In the studies considered in this meta-analysis, most patients initiated a first-line based on a 3TC-d4T backbone with either EFV or NVP, which is no longer recommended. However, in the most recent WHO guidelines the strategy hasn’t changed (two NRTIs associated to one NNRTI) [28], and new regimens are unlikely to have a higher genetic barrier to resistance. We did not find evidence that the incidence rate of switching was different according to the first-line used. Drug choices probably only partly explain the incidence rate of switching to second-line ART.

In the studies considered, second-line ART was identified as solely a PI introduction, or as a PI-introduction along with a change of at least one NRTI. When the NRTI-backbone was not modified, one can expect some changes to be toxicity-related rather than related to failure. However, this would mean that the incidence rate of switching for therapeutic failure may then be even lower than the incidence rate estimated in this meta-analysis.

Current guidelines recommend that an individual is on ART for at least 6 months before it can be determined that a regimen has failed [28] and biological monitoring (CD4 count and viral load when available) is recommended every 6 months. Therefore, switching to second-line ART is expected to occur after 12 months of first-line ART. The number of virological failure is also likely to increase as the time spent under-first-line ART increases. In this meta-analysis, the mean duration of follow-up ranged from 12 to 20 months in 7 of the 9 studies considered. The studies considered in the meta-analysis tended to report switches to second-line ART that occurred during the first few years on ART. This could explain why we did not find that the incidence rate was different between programmes with and without viral load monitoring.

The low incidence rate of switching to second-line ART and the poor prognosis of patients under second-line ART suggest that the monitoring of patients under ART is critical and efforts should be made to make viral loads more widely accessible, to improve the information system and logistics, to help clinicians use viral load measurements in their clinical practice, but also to improve access to second- and third-line molecules in resource-limited settings.

## Supporting Information

Checklist S1 **PRISMA Checklist.**
(DOC)
