# Correction: Persistent Difficulties in Switching to Second-Line ART in Sub-Saharan Africa — A Systematic Review and Meta-Analysis

**DOI:** 10.1371/journal.pone.0095820

**Published:** 2014-04-15

**Authors:** The PLOS ONE Staff

Figure 2 is incorrect. The authors have provided a corrected version here.

**Figure 2:**
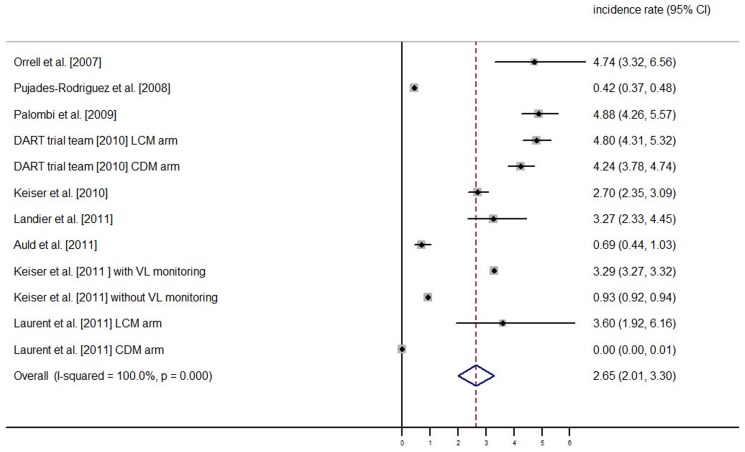
Incidence rate of switching to second-line ART (expressed per 100 person-years) – Estimation from 9 studies providing 11 incidences of switching to second-line ART.
